# Neuromedins U and S involvement in the regulation of the hypothalamo–pituitary–adrenal axis

**DOI:** 10.3389/fendo.2012.00156

**Published:** 2012-12-05

**Authors:** Ludwik K. Malendowicz, Agnieszka Ziolkowska, Marcin Rucinski

**Affiliations:** Department of Histology and Embryology, Poznan University of Medical SciencesPoznan, Poland

**Keywords:** neuromedin U, neuromedin S, hypothalamus, pituitary, adrenal

## Abstract

We reviewed neuromedin U (NMU) and neuromedin S (NMS) involvement in the regulation of the hypothalamo–pituitary–adrenal (HPA) axis function. NMU and NMS are structurally related and highly conserved neuropeptides. They exert biological effects via two GPCR receptors designated as NMUR1 and NMUR2 which show differential expression. NMUR1 is expressed predominantly at the periphery, while NMUR2 in the central nervous system. Elements of the NMU/NMS and their receptors network are also expressed in the HPA axis and progress in molecular biology techniques provided new information on their actions within this system. Several lines of evidence suggest that within the HPA axis NMU and NMS act at both hypothalamic and adrenal levels. Moreover, new data suggest that NMU and NMS are involved in central and peripheral control of the stress response.

## Introduction

In search for new biologically active peptides, the group of Minamino, Kangawa, and Matsuo in the 1980s isolated numerous small neuropeptides from porcine spinal cord. All of them exerted potent smooth-muscle stimulating activity. These short peptides have been named neuromedins (Minamino et al., 1985). Their sequences and biological activities are similar to some known neuropeptides and therefore they are commonly divided into four groups (classes):

Bombesin-like neuromedins—neuromedin B (NMB) and neuromedin C (NMC) (Minamino et al., 1983, 1984b).

Kassinin-like neuromedins—neuromedin K (NMK) and neuromedin L (NML) (Kangawa et al., 1983; Minamino et al., 1984a).

Neurotensin-like neuromedins—neuromedin N (NMN) (Minamino et al., 1984c).

Neuromedins U (NMU), for which no substantial homology with other known neuropeptides was found (Minamino et al., 1985). This group, however, was expanded in 2005, when Mori et al. (2005, 2008) isolated neuromedin S (NMS) from rat brain. NMS is composed of 36 amino acid residues and both peptides share the same amidated C-terminal heptapeptide. Furthermore, both NMU and NMS appeared to be endogenous ligands for the orphan G protein-coupled receptors FM-3/GPR66 and FM-4/TGR-1, identified earlier as type-1 and type-2 NMU receptors (NMUR1 and NMUR2), respectively (Tan et al., 1998; Howard et al., 2000; Raddatz et al., 2000; Mori et al., 2005).

Identification of specific NMU receptors (NMUR1 and NMUR2) and its anorexigenic action have enhanced interest in physiological role of NMU and NMS (Howard et al., 2000; Ida et al., 2005). Advances in these studies were recently reviewed (Brighton et al., 2004; Mori et al., 2008; Mitchell et al., 2009a; Budhiraja and Chugh, 2009). Present review, on the other hand, will focus on expression and of role of NMU/NMS system in hypothalamo–pituitary–adrenal (HPA) axis functioning, updating thus our earlier reviews (Malendowicz and Markowska, 1994; Malendowicz, 1998; Malendowicz et al., 2013).

## Isolation, structure, and synthesis of NMU and NMS

**Figure 1:**
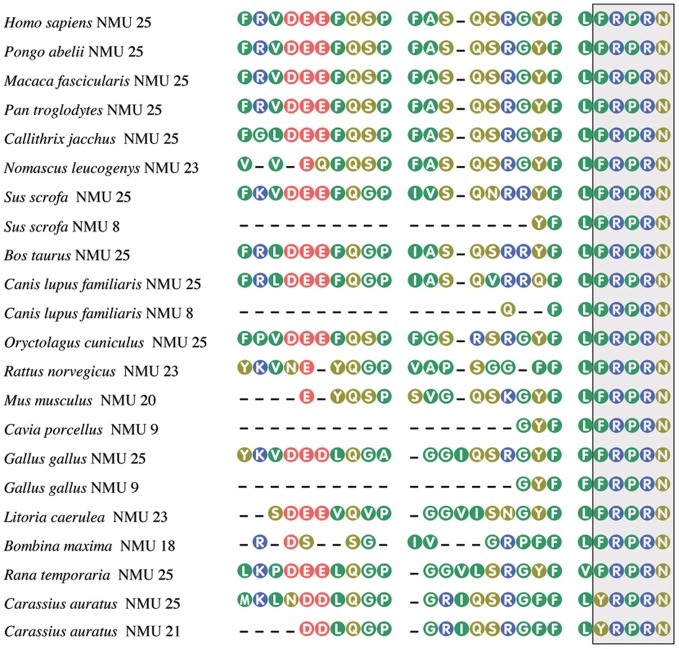
**Amino acid sequences of neuromedin U from some mammalian, avian, piscine, and amphibian species.** The box, highlighting the C-terminal pentapeptide, shows conservation of this sequence in vertebrates, except of goldfish. From Mitchell et al. (2009a), modified. Amino acid sequences were acquired from NCBI. Numbers after NMU denote peptide length.

Originally NMU was isolated from porcine spinal cord in two molecular forms, one containing 25 (NMU25) and the other 8 (NMU8) amino acid residues (Minamino et al., 1985). Subsequently NMU was isolated from other vertebrates, among them humans (Austin et al., 1995), rat (Conlon et al., 1988), guinea pig (Murphy et al., 1990), dog (O'Harte et al., 1991a), rabbit (Kage et al., 1991), chicken (O'Harte et al., 1991b; Domin et al., 1992), frogs—*Rana temporaria*, *Litoria caerulea*, and *Bombina maxima* (Domin et al., 1989b; Salmon et al., 2000; Lee et al., 2005), and goldfish (Maruyama et al., 2008). Amino acid sequences of NMU from different species are shown in Figure 1.

In the mammalian NMUs a common C-terminal sequence—Phe-Leu-Phe-Arg-Pro-Arg-Asn-NH_2_—contains the active site of the neuropeptide, which is formed by the amino acid residues between positions 2 and 8 (Hashimoto et al., 1991; Sakura et al., 1991). Recent data indicate that in most species studied, the five amino acids at the C-terminus of the NMUs are totally conserved, suggesting that this region is of major importance for biological activity (Lo et al., 1992; Brighton et al., 2004; Mitchell et al., 2009b). Amidation of the C-terminus of NMU is required for receptor activation (for review see Brighton et al., 2004).

**Figure 2:**
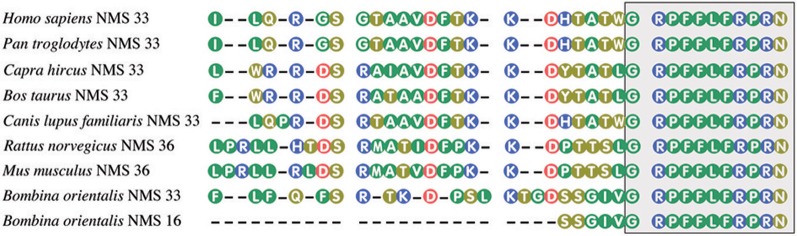
**Amino acid sequences of neuromedin S from some mammalian and amphibian species.** The box, highlighting the C-terminal decapeptide, shows conservation of this sequence in vertebrates. NMU and NMS share the same amidated C-terminal heptapeptide. Amino acid sequences were acquired from NCBI. Numbers after NMS denote peptide length.

Unexpectedly, in 2005, Mori et al. (2005) isolated from the rat brain a new 36 amino acid peptide related to NMU, which appeared to be another endogenous ligand of FM-4/TGR-1 receptor. This neuropeptide is highly expressed in the suprachiasmiatic nucleus of the hypothalamus and therefore was designed as NMS. Human NMS, on the other hand, is composed of 33 amino acid residues. All NMS share a C-terminal core structure with NMU. NMU and NMS share the same amidated C-terminal heptapeptide and bind to the same receptors NMUR1 and NMUR2. Amino acid sequences of NMS from different species are shown in Figure 2.

It should be emphasized that NMU and NMS genes are located on different chromosomes (NMU on 4q12 and NMS on 2q11.2) (Mori et al., 2005). Moreover, evidences indicate that during the process of evolution NMU and NMS genes had already diverged at the level of the Amphibia (Chen et al., 2006).

**Figure 3:**
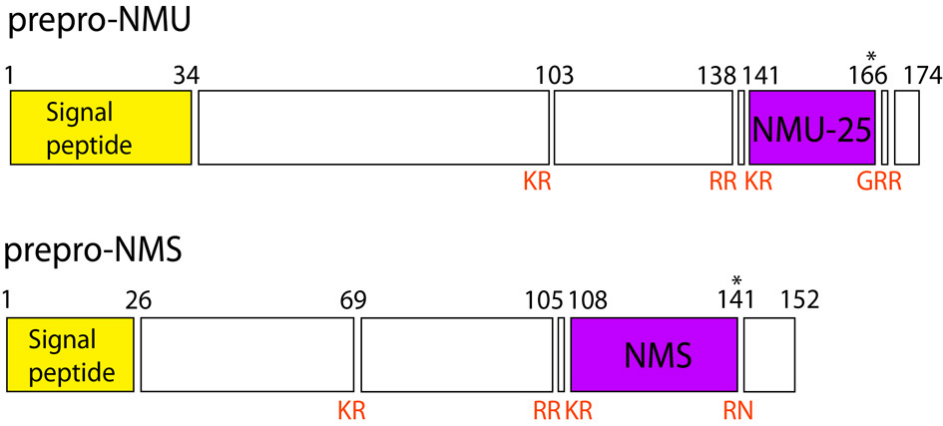
**Schematic structure of prepro-NMU and prepro-NMS in humans.** Data from Protein Knowledgebase (UniProtKB) P48645 and Q5H8A3, respectively. Schematic structure of prepro-NMU is modified from Austin et al. (1995). Numbers refer to residues and cleavage sites are given in red. Asterisk marks amidated asparagine.

In humans both NMU and NMS genes are composed of 10 exons and 9 introns. The mRNA lengths encoded by these genes are 816 and 485 bp, respectively. The exon–intron boundaries in the NMU and NMS prepro-proteins are comparably conserved (Mori et al., 2008). General structures of encoded prepro-NMU and prepro-NMS are shown in Figure 3.

## NMU and NMS receptors

Early studies revealed the presence of highly specific ^125^I-NMU binding sites on membranes prepared from the rat uterus. The binding was saturable and specific and Scatchard analysis suggested a single class of binding site with a K_d_ of 0.35 nM (Nandha et al., 1993).

By means of modern molecular biology techniques two receptors for NMU were identified. In 1998, Tan et al. (1998) cloned a GPCR (FM-3) from human and murine cDNA libraries. This DNA segment has homology to the growth hormone secretagogue receptor and the neurotensin receptor. Subsequently, by means of a “reverse pharmacological” method NMU was identified as an endogenous ligand for the orphan human GPCR, FM-3 (or GPR66) (Fujii et al., 2000; Hedrick et al., 2000; Hosoya et al., 2000; Howard et al., 2000; Kojima et al., 2000; Raddatz et al., 2000; Shan et al., 2000; Szekeres et al., 2000). After identification of the second NMU receptor, the first one had been named NMUR1.

The NMUR2 (FM-4, TGR1) gene, on the other hand, was identified based on its sequence similarity with NMUR1 (Hosoya et al., 2000; Howard et al., 2000; Raddatz et al., 2000; Shan et al., 2000). NMUR1 gene is located on human chromosome 2—position q37.1 and NMUR2 gene on chromosome 5—position q33.1 (Mitchell et al., 2009a).

**Figure 4:**
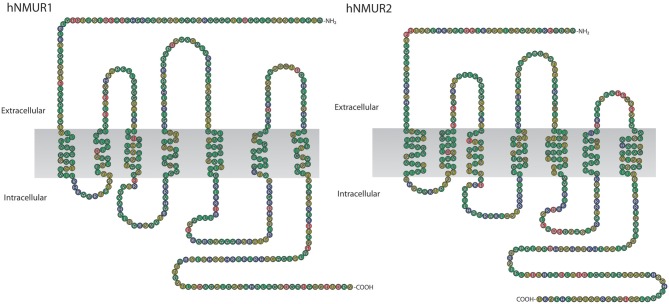
**Schematic representation of human NMUR1 and NMUR2 receptors.** NMUR1 receptor is composed of 426 amino acid residues while NMUR2 of 415. Amino acid sequences were acquired from NCBI, accession numbers AAG24793.1 and EAW61653.1, respectively.

In humans NMUR1 gene consists of 3 exons and 2 introns, the size of encoded mRNA is 3274 bp and the receptor is composed of 426 aa residues. NMUR2 gene, on the other hand, consists of 4 exons and 3 introns. The size of its mRNA is 2067 bp and the receptor is composed of 415 aa residues (Figure 4).

NMS has also been identified as an endogenous ligand of NMUR1 and NMUR2 receptors and some data indicate that NMUR2 has greater affinity to NMS than NMU (Mori et al., 2005).

Interaction of NMU and NMS with their receptors results in intracellular calcium mobilization and subsequent stimulation of inositol phosphates. These effects are mediated by both G_q/11_ and G_i/0_ proteins (Raddatz et al., 2000; Shan et al., 2000; Szekeres et al., 2000; Funes et al., 2002; Mori et al., 2005; Maruyama et al., 2011). Moreover, both neuromedins are also able to activate the mitogen activated protein kinase ERK1 and ERK2 (Brighton et al., 2004).

## Expression of NMU and NMS and their receptors in the hypothalamo–pituitary–adrenal axis

### Hypothalamus

#### NMU and NMS

Soon after NMU identification, high concentrations of NMU-like immunoreactivity were found in extracts of the rat, mouse, and human hypothalamus (Domin et al., 1986, 1988). In rat hypothalamus concentration of NMU-like immunoreactivity was reported as 31.2 ± 5.6 pmol/g (nearly 6 times higher than in the anterior pituitary lobe). Subsequent immunohistochemical studies demonstrated the presence of NMU-like substances in the hypothalamic paraventricular (PVN) and arcuate nucleus of the rat (Honzawa et al., 1987; Ballesta et al., 1988; Steel et al., 1988). On the other hand, molecular biology techniques demonstrated low to moderate levels of NMU mRNA in rat (RT-PCR) and human (QPCR) hypothalamus (Fujii et al., 2000; Szekeres et al., 2000). Single-cell reverse transcription-multiplex polymerase chain reaction (single-cell RT-mPCR) technique revealed that 14.7% parvocellular neurons of the rat PVN expressed NMU mRNA (Chu et al., 2012).

NMU mRNA is present in hypothalamus of WT mice and, in contrast, NMU mRNA could not be detected in NMU KO mice (Fukue et al., 2006).

In the frog (*Rana esculenta*) NMU-like immunoreactivity was observed in perikaria of the dorsal nucleus of the hypothalamus and the caudal part of the infundibulum (Maderdrut et al., 1996). Although authors emphasize that this pattern of immunoreactivity distribution differs notably from that seen in mammals, it is necessary to emphasize that the ventral infundibular nucleus of the frog is homologous to the mammalian arcuate nucleus (Neary and Northcutt, 1983; Ten Donkelaar, 1998).

The highest expression of NMS mRNA was found by RT-PCR in rat hypothalamus (Mori et al., 2005). Subsequently, *in situ* hybridization demonstrated that this neuropeptide was specifically expressed in the hypothalamic suprachiasmatic nuclei (SCN). In the rat hypothalamus expression of NMS gene was nearly 3-fold higher than that of NMU gene (Rucinski et al., 2007). During postnatal development levels of NMS mRNA attained maximum at prepubertal stage and adulthood (Vigo et al., 2007).

#### NMU receptors

Earliest studies revealed that NMUR1 is expressed predominantly in periphery while NMUR2 in the central nervous system (for review see Brighton et al., 2004; Mitchell et al., 2009a). In agreement with this principle in the rat hypothalamus, by means of QPCR Raddatz et al. (2000) demonstrated high expression of NMUR2 and a notably lower one of NMUR1. This finding was confirmed by numerous groups (Fujii et al., 2000; Howard et al., 2000; Qiu et al., 2005; Rucinski et al., 2007; Vigo et al., 2007). NMUR2 gene is also expressed in the PVN. By means of the single-cell RT-mPCR analysis (multiplex) Qiu et al. (2005) demonstrated that NMU-sensitive PVN neurons abundantly expressed NMUR2 mRNA but expressed NMUR1 mRNA to a lesser extent or not at all.

Detailed mapping of NMUR2 mRNA expression in the rat brain by *in situ* hybridization revealed the most intense signal in ependymal cells of the third ventricle and moderate signal in the PVN (Guan et al., 2001; Graham et al., 2003). Presence of NMUR2 receptors in these regions of the brain had also been confirmed by receptor autoradiography (Mangold et al., 2008).

Similar pattern of NMU receptor expressions is observed in human hypothalamus, with high expression of NMUR2 and a negligible one of NMUR1 (Szekeres et al., 2000; Gartlon et al., 2004).

### Pituitary gland

#### NMU and NMS

High concentrations of NMU-like immunoreactivity were found in the pituitary gland of the rat as early as in 1987 (Domin et al., 1987, 1988, 1989a). In rat anterior lobe concentration of NMU-like immunoreactive substances was 6.3 pmol/gland (mean) while in posterior lobe 0.3 pmol/gland only. Furthermore, Northern blot analysis using total RNA extracted from rat anterior pituitary demonstrated high levels of NMU mRNA in the gland (Lo et al., 1992).

High concentrations of NMU protein in rat pituitary gland are accompanied by high expression of NMU gene (Fujii et al., 2000). High expression of this gene in anterior pituitary lobe of the rat was confirmed by QPCR (Rucinski et al., 2007; Shimizu et al., 2008). It should be emphasized that NMU mRNA is also expressed in cultured pituitary cells of the rat. Using quantitative *in situ* hybridization, high concentration of NMU transcripts was also found in part tuberalis of the rat pituitary (Ivanov et al., 2002; Nogueiras et al., 2006).

Immunohistochemistry demonstrated the presence of NMU-immunoreactive substances in the intermediate and the anterior pituitary gland lobes of mouse, rat, and human (Ballesta et al., 1988; Steel et al., 1988). Electron microscopy studies revealed NMU-like immunoreactivity in some thyrotropes and most corticotropes of the rat pituitary gland. NMU is colocalized with galanin and ACTH in the same secretory granules (Cimini et al., 1993; for review see Malendowicz, 1998; Cimini, 2003).

In developing rat NMU-immunopositive cells appear in anterior pituitary at day E15 (Cimini, 2003). Their number increases at day E20 and then decreases. More than 60% of NMU-immunoreactive cells contain ACTH at E15, and, after falling to about 40% at E16, this value is more or less constant until E21.

NMU mRNA is expressed in pituitary gland of WT mice, but in contrast, NMU mRNA could not be detected in NMU KO mice (Fukue et al., 2006).

Expression of NMS gene in rat pituitary gland was found by Mori et al. (2005). In rat anterior pituitary expression level of NMS gene however, was notably lower than that of NMU gene (Rucinski et al., 2007).

#### NMU receptors

Conflicting data were reported on expression of NMUR1 and NMUR2 in pituitary gland. By means of QPCR low expression of both receptors in human pituitary gland was reported by Raddatz et al. (2000). These data were confirmed by other groups (Shan et al., 2000; Gartlon et al., 2004).

The earliest studies did not reveal NMUR1 gene expression in the rat pituitary gland while that of NMUR2 was very low (Fujii et al., 2000; Gartlon et al., 2004). On the other hand, in rat adenohypophysis expression of NMUR1 gene, but not of NMUR2 was observed by our group (Rucinski et al., 2007).

Expression of both NMUR1 and NMUR2 genes was observed in mouse pituitary gland of both WT and NMU KO mice (Fukue et al., 2006).

### Adrenal gland

Only scanty data are available on expression of NMU–NMS and their receptors in adrenal glands. Very low levels of NMU mRNA in the rat adrenal were reported by Fujii et al. (2000). Detailed studies on rat adrenal gland revealed very low expression of NMU and NMS genes at mRNA levels (Rucinski et al., 2007).

In human adrenal gland both NMUR2 mRNA (PCR) and protein (dot blot method) were identified in 2000 (Raddatz et al., 2000; Shan et al., 2000).

In the rat adrenal gland NMUR2 mRNA could not be demonstrated (Hosoya et al., 2000; Fujii et al., 2000). In immature rat adrenal NMUR1 was expressed at both mRNA and protein (immunohistochemistry) levels while the signal from NMUR2 was absent. NMUR1 mRNA was detected in all adrenocortical zones and in medulla of the gland (Ziolkowska et al., 2008). Moreover, the presence of NMUR1 mRNA in isolated zona glomerulosa and fasciculata/reticularis cells rules out the possibility that the expression was due to the presence in the specimens assayed of the non-parenchymal components of the gland. Expression of NMUR1 as mRNA and protein was demonstrated in adrenal gland of intact rat, in enucleation-induced regenerating gland, in hemiadrenalectomized animals (compensatory adrenal growth) as well as in ACTH-stimulated one (Trejter et al., 2008, 2009; Malendowicz et al., 2009).

## NMU and NMS in the hypothalamo–pituitary–adrenal axis functioning

### Hypothalamus

The above described localization of elements of NMU/NMS and NMUR2 system in hypothalamus forms a base of regulation by NMU and NMS of HPA axis functioning.

The earliest experiments with intracerebroventricular (icv) injection of NMU demonstrated a strong increase in Fos-immunoreactive nuclei in the PVN and supraoptic nucleus (SON) of the rat hypothalamus (Niimi et al., 2001; Ozaki et al., 2002). Almost all CRH-containing neurons in the parvocellular divisions of the PVN expressed Fos-like immunoreactivity 90 min after icv administration of NMU (Yokota et al., 2004).

Subsequent studies demonstrated direct NMU effects on CRH and arginine vasopressin (AVP) release by rat hypothalamic explants *in vitro* (Wren et al., 2002). At the 100 nM NMU concentration CRH and AVP release was nearly doubled when compared to controls. Moreover, results of whole cell patch-clamp recordings revealed that NMU directly depolarized the subpopulation of PVN parvocellular, but not magnocellular neurons via enhancement of the hyperpolarization-activated inward current (Qiu et al., 2003). Direct effects of both neuromedins on PVN was also suggested by *in vitro* electrophysiological studies which showed that NMU and NMS increased the neuronal firing rates in both arcuate and PVN nuclei slices (Nakahara et al., 2010).

NMU also regulates HPA axis in birds. Icv administration of NMU in chicks significantly upregulated mRNA expression of CRH in the hypothalamus (Kamisoyama et al., 2007).

NMS likewise affects CRH neurons in PVN. In the rat icv administration of this neuromedin increased POMC mRNA expression in the arcuate nucleus and CRH mRNA in the PVN (Ida et al., 2005; Nakahara et al., 2010). NMS notably stimulated Fos-immunoreactive cells in both hypothalamic nuclei. NMS also significantly increased firing rate of PVN cells. Stimulating effects of NMS on Fos-immunoreactive cells of the PVN was confirmed by another group (Sakamoto et al., 2007, 2008).

### Pituitary

Expression of NMUR1 in pituitary gland and colocalization of NMU and ACTH in pituitary corticotropes suggest NMU and NMS involvement in regulation of ACTH secretion. Regarding this, there is a growing body of evidence that in the rat NMU administered icv, into PVN or subcutaneously increases blood ACTH concentrations, via stimulation of CRH release.

First reports demonstrated that a single sc injection of NMU8 resulted in a transient increase in ACTH blood concentration while after 2–6-day treatment (low NMU8 dose) blood ACTH level remained unchanged (Malendowicz et al., 1993, 1994b; Trejter et al., 2009). Only the higher dose of NMU8 (6 μg/100 g/day for 6 days) increased the level of circulating ACTH.

In the rat unilateral adrenalectomy notably increased plasma ACTH concentrations and NMU administration (sc) into hemiadrenalectomized rats did not significantly change corticotropin levels (Malendowicz et al., 2009).

In the rat icv administration of NMU (0.1, 1, and 3 nmol/rat) resulted in a dose-dependent increase of plasma ACTH concentrations, an effect significantly reduced by pretreatment with anti-NMU IgG (Ozaki et al., 2002). Similar dose-dependent effects of NMU on plasma ACTH concentrations were found after iPVN neuropeptide administration (Wren et al., 2002). Again, chronic iPVN NMU administration (twice-daily of 0.3 nmol NMU for 7 days) did not change plasma ACTH concentrations (Thompson et al., 2004). Only one group was unable to demonstrate stimulating effect of icv administered NMU on plasma ACTH concentrations in the rat (Rokkaku et al., 2003).

Unfortunately, no studies have as yet investigated direct effects of NMU and NMS on pituitary ACTH secretion. Our preliminary data indicate that neither NMU nor NMS affect ACTH release by quarters of the rat adenohypophysis, while the response to CRH was normal. This observation may suggest that observed *in vivo* stimulating effect of NMU/NMS on ACTH secretion is mediated via hypothalamus.

### Adrenal

Potent stimulating effects of exogenous NMU on adrenocortical steroid secretion in the rat have been described as early as in 1993. A single sc injection of NMU resulted in a transient increase in ACTH blood concentration (between 3 and 12 h) and a sustained (24 h) elevation of plasma corticosterone concentration (Malendowicz et al., 1993, 1994b). These data demonstrated stimulating effect of neuropeptide on adrenal cortex, possibly partially due to the direct effect of NMU on the gland.

In subsequent searches for mode of NMU action on corticosteroid secretion our group found that NMU had no effect on basal and ACTH-stimulated corticosterone secretion by freshly isolated or cultured inner zone adrenocortical cells, nor did it change their cytosolic Ca^2+^ concentration (Malendowicz et al., 1994b; confirmed by Ziolkowska et al., 2008). However, this neuropeptide stimulated corticosterone output by adrenal slices, but not by fragments of adrenocortical autotransplants lacking medullary chromaffin cells (Malendowicz et al., 1994a). Detailed analysis revealed that at all concentrations tested NMU increased basal pregnenolone and total post-pregnenolone steroid output by gland slices containing both cortex and medulla. The increase in total post-pregnenolone steroid output induced by low concentrations of NMU8 was due to similar rises in the production of non-18-hydroxylated steroids; conversely, that provoked by higher concentrations of the peptide was almost exclusively caused by the rise in the yield of 18-hydroxylated steroids. The stimulating effect of NMU8 on pregnenolone output was blocked by both alpha-helical-CRH and corticotropin-inhibiting peptide, which are competitive inhibitors of CRH and ACTH, respectively. These data suggest that NMU affects corticosteroid secretion indirectly by acting on the medullary chromaffin cells, which in turn may paracrinally stimulate cortex of the gland. We also suggested that the medullary mediator of NMU action on adrenal cortex may activate 18-hydroxylation and aldosterone synthase activity. On the other hand, we cannot rule out the possibility of NMU action on adrenal cortex via stimulation of adrenaline secretion by adrenal medulla. Recently it has been demonstrated that centrally administered NMU evokes secretion of adrenaline from the adrenal medulla (Sasaki et al., 2008). Medullary adrenaline may in turn modulate secretion of corticosteroids (for review see Nussdorfer, 1996; Bornstein et al., 1997; Ehrhart-Bornstein et al., 1998).

Stimulating effects of NMU on corticosteroid secretion also were observed after icv or iPVN neuropeptide administration. In the rat acute iPVN administration of NMU dose-dependently increased plasma corticosterone concentrations (Wren et al., 2002). Similar effect was observed after icv NMU administration (Ozaki et al., 2002). In contrast, another group reported lack of icv NMU administration effects on plasma corticosterone levels (Gartlon et al., 2004).

Stimulating effects of icv administered NMU on plasma corticosterone levels were confirmed by experiments with anti-NMU IgG (Jethwa et al., 2006). In rats administration of anti-NMU IgG significantly attenuated the dark phase rise in plasma corticosterone concentrations.

In contrast to acute administration, chronic iPVN administration of NMU produced an elevation of plasma corticosterone levels while plasma ACTH concentrations remained unchanged (Thompson et al., 2004). Thus, in chronically NMU administered rats elevated plasma corticosterone levels, not accompanied by increased ACTH concentrations, were independent of the mode of NMU administration (sc vs iPVN) (Malendowicz et al., 1993, 1994b; Thompson et al., 2004).

The stimulating effect of sc administered NMU on plasma corticosterone concentrations also was found in rats during enucleation-induced regeneration, as well as in rats treated with low ACTH doses (Trejter et al., 2008, 2009). On the other hand, NMU8 administration to immature rats was found to raise aldosterone, but not corticosterone concentrations (Ziolkowska et al., 2008).

NMS, like NMU also stimulates corticosteroid secretion. In rats icv NMS administration resulted in nearly 5-fold increase in plasma corticosterone concentrations and the effect was dependent on neuropeptide dose (Jászberényi et al., 2007). Likewise, in castrated Holstein steers both NMS and NMU evoked a dose-related increase in plasma cortisol concentrations (Yayou et al., 2009). Of interest is that also in chicks icv NMS administration stimulated corticosterone release (Tachibana et al., 2010).

In contrast to the above described NMS effects on corticosterone/cortisol secretion, an opposite effect was seen in Rhesus monkeys (Jahan et al., 2011). In their experiments NMS was infused through a teflon cannula implanted into saphenous vein. Blood samples were collected 45 min before and 120 min after NMS administration at 15 min intervals. Under these conditions NMS resulted in a significant decrease in plasma cortisol levels at 40 and 60 nmol doses while a non-significant difference was observed at 20 nmol. This unexpected finding may suggest that in non-human primates NMS exerts inhibitory effect on HPA axis functioning. However this finding remains to be confirmed.

Recent data also demonstrated direct stimulating effect of NMU on proliferative activity of immature rat inner adrenocortical cells in primary culture (Ziolkowska et al., 2008). Moreover, in enucleation-induced adrenal regeneration NMU notably enhanced proliferative activity of adrenocortical cells (Trejter et al., 2008). A similar stimulating effect of sc administered NMU on proliferative activity (metaphase index) was seen in ACTH treated rats (Trejter et al., 2009). In contrast, in hemiadrenalectomized rats NMU notably inhibited adrenocortical cell proliferation in both zona glomerulosa and zona fasciculata, as assessed by the metaphase index (Malendowicz et al., 2009). Since these effects were independent of changes in blood ACTH, they could reflect an interaction of NMU with the neural system innervating the adrenal gland, which is responsible for compensatory adrenal growth.

## Involvement of NMU and NMS in the stress response

As it follows from the above presented data, NMU and NMS are linked to the HPA axis functioning. Independently of mode of administration (icv, iPVN, or sc) NMU activates CRH containing neurons and stimulates CRH secretion, which in turn triggers pituitary ACTH and adrenal corticosterone/cortisol secretion. In this regard it is not astonishing that NMU and NMS are involved in central and peripheral control of the stress response. These studies were initiated by Hanada et al. (2001) who observed stress-related behavior (gross locomotor activity, face washing and grooming) in icv NMU administered rats. This response was attenuated by pretreatment with alpha-helical CRH (antagonist of CRH) or anti-CRH IgG. Furthermore, NMU-induced increases in oxygen consumption and body temperature were attenuated in CRH KO mice. Subsequent studies demonstrated that blood corticosterone levels were significantly increased after 10 min of immobilization stress in wild-type mice, but not in NMU KO mice (Nakahara et al., 2004). Excessive grooming induced by icv NMU administration were abolished in the NMUR2 KO mice, an observation suggesting that NMUR2 plays a decisive role in stress/anxiety induction (Zeng et al., 2006).

Stress related behavior induced by icv NMU or NMS administration was also observed in cattle (Yayou et al., 2009). Administration of both neuropeptides tended to shorten the duration of lying and increase the number of head shaking in studied cattle. These behavioral changes were accompanied by increased levels of plasma cortisol.

## Concluding remarks

The above reviewed data clearly demonstrate NMU and NMS involvement in regulation of HPA axis growth and functions. However, their mechanism of action is far from being completely understood. The available experimental data suggest that within the HPA axis these neuromedins may exert both endocrine and/or paracrine/autocrine effects on target cells.

Endocrine effects of discussed neuropeptides require their release into the bloodstream. Only scanty data are available on NMU and NMS presence in general circulation. Manufacturers' manuals for various kits contain data on NMU and NMS concentration in plasma and comparable figures are shown in clinical studies (Ketterer et al., 2009). However, experimental data suggest that neither endogenous NMU nor NMS are circulating, at least in the rat (Peier et al., 2009). Furthermore, in general circulation the biological half-life of NMU is shorter than 5 min.

At the hypothalamus level the actions of NMU and NMS are rather well documented. Stimulation of CRH, arginine-vasopresssin and oxytocin release both *in vivo* and *in vitro* activates HPA axis and apart from elevation of ACTH and corticosterone/cortisol secretion, triggers behavioral parameters typical for stress reaction.

More puzzling is the effect of NMU on pituitary gland. As discussed earlier, in pituitary gland high levels of NMU are present, and the gland contains high concentration of NMU-like immunoreactive substances and is provided with NMUR1 receptor. NMU is colocalized with ACTH and numerous NMU-like immunoreactive cells are present in human extrapituitary corticotropinomas (Steel et al., 1988). Denef (2008) suggests that in the pituitary NMU may paracrinally regulate function of adjacent cells and a similar mode of action may be present in extrapituitary corticotropinomas. So far, direct effects of NMU/NMS on pituitary ACTH secretion have not been reported. Our preliminary data indicate that neither NMU nor NMS affect ACTH release by quarters of the rat adenohypophysis. It remains to be established whether inability of NMU and NMS to directly stimulate ACTH release from pituitary gland may be caused by possible high receptor occupancy by endogenous NMU.

In view of these findings, questions arise concerning the possible role of NMU in pituitary tumor formation. In their review on pituitary tumorogenesis Korbonits et al. (2004) mention NMU only as a potential messenger in the anterior pituitary. Thus, the possible role of NMU in development of pituitary and extrapituitary corticotropinomas requires further investigation. As far as endocrine system is concerned, an elevated expression of NMU gene was found in human ovarian cancer cell lines (Euer et al., 2005).

The available experimental data suggest that at the adrenal level NMU affects the steroid secretion indirectly by acting on the medullary chromaffin cells. On the other hand some data suggest direct effect of neuromedin on proliferation and growth of rat adrenocortical cells.

Thus, although great progress has been made in understanding NMU and NMS action within the HPA axis during the past years, much remains to be learned about their mechanisms of action within this system. Recent development of a metabolically stable analog of NMU, based on derivatization of the native peptide with high molecular weight poly(ethylene) glycol (PEG) (“PEGylation”) may be helpful in these attempts (Ingallinella et al., 2012).

### Conflict of interest statement

The authors declare that the research was conducted in the absence of any commercial or financial relationships that could be construed as a potential conflict of interest.

## Abbreviations

CRH: corticotropin releasing hormone
GPCR: G protein coupled receptor
HPA: hypothalamo–pituitary–adrenal
icv: intracerebroventrical
iPVN: intraparaventricular nucleus
KO: knock out
NMS: neuromedin S
NMU: neuromedin U
NMUR1: neuromedin U receptor 1
NMUR2: neuromedin U receptor 2
PCR: polymerase chain reaction
PVN: paraventricular nucleus
QPCR: quantitative real time polymerase chain reaction
RT-PCR: reverse transcription PCR
sc: subcutaneously
WT: wild type.
